# Acupotomy Alleviates Energy Crisis at Rat Myofascial Trigger Points

**DOI:** 10.1155/2020/5129562

**Published:** 2020-02-28

**Authors:** Yi Zhang, Ning-Yu Du, Chen Chen, Tong Wang, Li-Juan Wang, Xiao-Lu Shi, Shu-Ming Li, Chang-Qing Guo

**Affiliations:** ^1^School of Acupuncture-Moxibustion and Tuina, Beijing University of Chinese Medicine, Beijing 100029, China; ^2^Center for Early Childhood Development, Shijiazhuang Maternal and Child Health Care Hospital, Shijiazhuang 050051, China; ^3^Beijing Key Laboratory of TCM Basic Research on Prevention and Treatment of Major Disease, Experimental Research Center, China Academy of Chinese Medical Sciences, Beijing 100700, China; ^4^Department of Pain Medicine, Beijing Hospital of Traditional Chinese Medicine, Capital Medical University, Beijing 100010, China

## Abstract

The aim of this study was to determine the effects of acupotomy on energy crises in rat trigger points (TrPs) by measuring mechanical pain thresholds (MPTs) and levels of acetylcholinesterase (AChE), free sarcoplasmic calcium (Ca^2+^), adenosine 5′-triphosphate (ATP), adenosine 5′-monophosphate (AMP), substance P (SP), and calcitonin gene-related peptide (CGRP) in rat muscle TrP tissue. Male Sprague Dawley rats (*n* = 32) were randomly divided into four groups: control, TrP, acupotomy, and lidocaine injection. Enzyme-linked immunosorbent assays were used to measure AChE, and free sarcoplasmic Ca^2+^ concentrations were determined by fluorescent staining with Fura-2 AM; high-performance liquid chromatography was used to measure ATP and AMP, and SP and CGRP were evaluated by immunohistochemistry. Compared with the control group, free sarcoplasmic Ca^2+^, AMP, SP, and CGRP were higher in the model group, while MPT, AChE, and ATP were lower. Treatment with acupotomy or lidocaine injection reduced free sarcoplasmic Ca^2+^, SP, and CGRP and increased MPTs and AChE levels compared with the model group. However, only acupotomy also led to decreased AMP and increased ATP levels relative to the model group. We conclude that acupotomy can alleviate energy crises at TrPs.

## 1. Introduction

Myofascial pain syndrome (MPS) is a group of clinical disorders characterized by chronic pain arising from soft tissue, associated with one or multiple trigger points (TrPs) [1]. Myofascial TrPs are irritable points characterized by taut skeletal muscle bands. They can cause soft tissue pain that is activated by pressing, pulling, or overuse. TrPs can contribute to local pain, referred pain, and local twitch responses. They can also decrease muscle strength, work endurance, and coordination as well as cause other types of muscle dysfunction. TrPs are also associated with autonomic nerve manifestations, such as hyperhidrosis and arrector pili muscle response. Among patients with soft tissue pain, 20%–95% suffer from MPS [2].

Although the exact nature of TrPs is unknown, Simons proposed the energy crisis hypothesis, which was subsequently developed to generate the integrated hypothesis [3, 4]. It contends that contributing factors lead to the malfunction of motor endplates and excessive acetylcholine (ACh) leaking from the endplates, causing continuous depolarization of muscle cell membranes and calcium (Ca^2+^) release from the sarcoplasmic reticulum, which cannot be reabsorbed. Therefore, the sarcoplasm continually has high Ca^2+^ concentrations, causing persistent muscle fiber contractions and the formation of taut intramuscular bands that can be palpated [5]. Continuous muscle contraction leads to local hypoxia and hypermetabolism, resulting in local energy crises and the release of sensitizing substances such as 5-hydroxytryptamine, histamine, bradykinin, and substance P (SP). This stimulates nerve endings, causing pain and sympathetic neuronal symptoms [6].

Acupotomy, also referred to as miniscalpel-needle acupuncture, is a fairly recent development in the ancient practice of acupuncture. Acupotomy involves a combination of acupuncture needle insertion and surgical incision and is effective for relieving TrPs [7]. While TrPs are common sites for acupotomy, the mechanism underlying the effects of acupotomy on TrPs remains unclear. In this study, mechanical pain thresholds (MPTs) and levels of acetylcholinesterase (AChE), free sarcoplasmic Ca^2+^, adenosine 5′-triphosphate (ATP), adenosine 5′-monophosphate (AMP), calcitonin gene-related peptide (CGRP), and SP were evaluated in rat TrPs, and the effects of acupotomy on these indicators were compared with the effects of local lidocaine injection. The results provide a theoretical basis for different interventions to treat TrPs, including needle insertion at ashi points as described in TCM.

## 2. Materials and Methods

### 2.1. Animals

Male specific pathogen-free Sprague Dawley rats (*n* = 32, 9 weeks old, weight 300 ± 15 g) were provided by Beijing Vital River Laboratory Animal Technology Co., Ltd. (animal batch number: SCXK(Jing)2016-0006). Rats were raised in the animal house at the Beijing University of Chinese Medicine with five rats per cage, free access to feed and water, natural lighting, indoor temperature of 22°C ± 2°C, humidity of 40%–60%, and regular ultraviolet disinfection. The 32 rats were randomly divided into 4 groups (*n* = 8 each; control, TrP model, acupotomy, and lidocaine injection) using the random number table method. During the experiment, the rats were treated and handled with strict adherence to the standards set by the Ethical and Animal Research Committees, with approval from the National Natural Science Foundation (no. 81503653).

### 2.2. Experimental Reagents and Equipment

Experimental materials included acupotomes (0.4 × 40 mm; ZYHY, Beijing, China), lidocaine hydrochloride injections (2%; Yookon, Beijing, China), normal saline (Yookon), iodophor (Tiangen, Kunming, China), and disposable sterile syringes (Yuekang, Changzhou, China). Other reagents used were rat AChE enzyme-linked immunosorbent assay (ELISA) kit (CSB-E11304r; Huamei, Wuhan), phosphate-buffered saline (PBS) solution (Hyclone, Logan, UT, USA), Dulbecco's modified Eagle's medium (DMEM; Hyclone), Krebs-Henseleit solution (in mmol/L: NaCl, 118.50; NaHCO_3_, 25.00; KCl, 4.70; MgSO_4_, 1.20; KH_2_PO_4_, 1.20; glucose, 11.00; CaCl_2_, 1.80; NaOH to pH 7.4), Fura-2 AM powder (F1221; Invitrogen), Fura-2 Calcium Imaging Calibration Kit (F6774; Invitrogen), 5′-adenosine triphosphate disodium salt hydrate (Sigma), sodium adenosine 5′- monophosphate (Sigma), rat SP immunohistochemical kits (ab14184, GR206575-1; Abcam), and rat CGRP immunohistochemical kits (ab81887, GR291095-3; Abcam).

### 2.3. Model Preparation

The rats, except those in the control group, were prepared as TrP models by applying “blunt striking injury and eccentric exercise” as previously described [5]. Briefly, the rats were anesthetized with 20% urethane solution injected into the abdominal cavity (0.5 mL/100 g). Rats were then fixed onto a plank under a striker, and the middle of the left vastus medialis was marked as the target. The striker was allowed to fall freely from a height of 20 cm to hit the marked position. On the second day, the rats were placed on a −16° treadmill set, so that they ran downhill continuously for 90 min, with the speed gradually rising to 16 m/min, once per week, for 8 weeks. After the 8th week, rats in the control and model groups were examined by electromyography to confirm TrP model establishment.

### 2.4. Interventions

Rats in the four experimental groups described above were treated as follows: (1) Control, no intervention; (2) TrP model, no intervention after model preparation; (3) Acupotomy, acupotomy was applied 1 week after TrP model preparation; and (4) Injection group, lidocaine injections given 1 week after model preparation (20% urethane solution injected into the abdominal cavity at a dosage of 0.5 mL/100 g, iodophor application to the TrP in the vastus medialis, and injection of 0.5 mL of 1% lidocaine into the taut band once a week for 3 weeks). Acupotomy intervention was conducted once per week for 3 weeks. The specific operation conducted at each point was as follows: (1) insertion of an acupotome into a TrP in the vastus medialis to pierce the taut band; (2) insertion of an acupotome perpendicular to the belly muscle, with the edge of the acupotome parallel to the taut band; and (3) the acupotome was then withdrawn and the point pressed for 30 s.

### 2.5. Index Assessments

#### 2.5.1. Electromyography

Electromyography assessments were performed in the control and model groups. Rats were anesthetized by intraperitoneal injection of 20% urethane (0.5 mL/100 g) and then fixed on a board. The skin was cut to expose the left vastus medialis, and an electrode (0.3 mm) was inserted into the tail as a reference electrode. For the control group, an electrode was slowly inserted into the middle of the vastus medialis, and another electrode was inserted longitudinally 3 mm away from the last electrode into the vastus medialis. Finally, the wound was sutured. The same procedure was performed for the TrP group, but local twitching was considered confirmation of insertion into the TrP. Electromyography recordings were performed when the muscle was at rest (Z2J-MB-NCC08; NCC, Shanghai, China).

#### 2.5.2. TrP MPTs

After the last intervention, 32 rats were anesthetized by 20% urethane. Anesthesia induction was confirmed by squeezing their tails using a power of 600 g [8] to see if it caused stable abdominal muscle contraction. The mechanical sensitivity of rat TrPs was assessed using the Von Frey mechanical pain stimulator (Ugo Basile, Genomio, Italy) to stimulate the TrP skin area until ipsilateral hind limb movement occurred [9]. The MPT was defined as the minimum stimulus intensity that caused the movement of the hind limbs. TrP sensitivity was assessed on days 1, 2, 3, 5, and 7, next week after the last intervention. For each measurement, MPTs were determined three times, with an interval of 1 min between each test, and the mean of the results was calculated.

#### 2.5.3. Sample Collection

Samples were collected after behavioral assessment. Rats were deeply anesthetized using 20% urethane and then fixed on an operating table with the left vastus medialis fully exposed. An experienced physician confirmed the position of the TrP in the vastus medialis by lightly pressing the taut band using the thumb or forefinger. To collect the samples, the vastus medialis was divided at the marked TrP, the fascia was carefully removed, and muscle tissue samples (1 × 0.2 cm) were excised. After sample collection, 32 rats were sacrificed by anesthesia overdose.

#### 2.5.4. AChE Elisa

Tissue samples were placed in 900 *μ*L PBS (pH 7.4), rapidly minced with ophthalmic scissors, and homogenized using an ultrasonic homogenizer (FS-100T; sxsonic, Shanghai, China) and vibration table (KJ-201C; Kangjian, Jiangsu, China). Tissue homogenates were centrifuged with 6,000 g at 4°C for 15 min, and the supernatants were separated. AChE levels in the samples were tested by an AChE ELISA kit according to the manufacturer's protocol. Optical density (OD) was read at 450 nm on a microplate reader (Multiskan MK3; Thermo Fisher Scientific, Waltham, MA, USA). Concentrations were calculated according to a standard curve, using the CurveExpert 1.3 software (https://www.curveexpert.net/).

#### 2.5.5. Fluorescent Staining to Detect Free Sarcoplasmic Ca^2+^

The basement membrane of isolated muscle tissue was carefully removed to expose the muscle fibers. Then, a 1 cm length of muscle tissue was cut along the direction of the muscle fibers and rinsed with PBS buffer to load with fluorescent dye. Fura-2 AM stock solution was prepared by dissolving Fura-2 AM powder (F1221, Invitrogen) in 20% Pluronic F127 DMSO solution. The muscle tissue was incubated with fluorescent staining solution (final Fura-2 concentration was 2.5 *μ*mol/L in DMEM solution) in the dark at the 37°C temperature. After 30 min loading, replace the fluorescent loading solution by KH solution and incubated another 5 min for twice at the 37°C temperature to remove unloaded Fura-2 AM. The fluorescence-stained samples in KH solution were then placed in 35 mm glass plates, and only the resting free sarcoplasmic Ca^2+^ in intact skeletal muscle fibers was measured using an Andor iXon3 EMCCD (Andor, Belfast, UK) with a 10x fluorescence objective lens. A Sutter DG4 Xenon lamp system (Sutter, Sacramento, CA, USA) was used as the excitation light source at 340 ± 10 nm and 380 ± 10 nm, separately, with emitted light measured at 510 ± 25 nm. Ca^2+^ images were collected from three different positions for each sample, and three muscle fibers were analyzed in each image. The mean value for the muscle fibers was considered the Ca^2+^ level in the tissue sample. Background fluorescence without staining was also measured for each sample. Fluorescence intensities at 340 and 380 nm were corrected by background intensity. The fluorescence intensity ratio was determined using the 340 and 380 nm excitation wavelengths, and tissue Ca^2+^ concentration was subsequently calculated using the following formula [10]: [Ca^2+^] = *K*_d_ × (*R*–*R*_min_)/(*R*_max_–*R*) × *S*_f2_/*S*_b2_ (in the formula, *K*_d_ is the effective dissociation constant, *R* is the fluorescence ratio, *S*_f2_ is the proportionality coefficient for free dye measured at wavelength *λ*_2_, and Sb_2_ is the proportionality coefficient for Ca^2+^-bound dye at *λ*_2_). The values of *R*_max_ and *R*_min_ were the ratio values measured by Fura-2 Calcium Imaging Calibration Kit (F6774, Invitrogen).

#### 2.5.6. ATP and AMP Measurement

Tissue samples were weighed and added to precooled 0.4 mol/L perchloric acid (5 *μ*L/mg) and then homogenized in an ultrasonic cell grinder (SCIENTZ-1200E; Scientz, Zhejiang, China). Homogenates were centrifuged at 10000 g (4°C, 10 min). Then, 100 *μ*L supernatant and 50 *μ*L perchlorate were added into a 1 mol/L potash-methanol mixture (volume 4 : 1), and the mixture was centrifuged at 10,000 g (4°C, 10 min). Next, a 100 *μ*L aliquot of the supernatant was removed, diluted with 100 *μ*L water, and assessed by high-performance liquid chromatography. The conditions were as follows: mobile phase, 30 mM NaH_2_PO_4_ (pH 6.25), 3% methyl alcohol; detection wavelength, 254 nm; sample size, 20 *μ*L; column temperature, room temperature; sample temperature, 4°C.

#### 2.5.7. Immunohistochemical Analysis of SP and CGRP

Muscle biopsies were fixed in 10% formalin and embedded in paraffin. After sectioned at 4 *μ*m thickness, sections were dehydrated in xylene, rehydrated in graded alcohol, and then rinsed with PBS. Then, the antigen was retrieved by microwave (800 W) in 0.01 M citric acid buffer (pH 6.0) for 10 min, washed with PBS three times, and then placed in the 3%H_2_O_2_ at room temperature for 60 min. The antibodies were diluted with PBS buffer. Slides were incubated (4°C) overnight with rabbit anti-SP (1 : 500; Abcam) or rabbit anti-CGRP (1 : 200; Abcam) and then with the appropriate secondary biotinylated antibody (Boster) for 60 min at room temperature. The slides were then incubated with the avidin-biotinylated enzyme complex and then put into a peroxidase reaction solution containing diaminoaniline. Afterward, fresh DAB was added. Then, the slides were washed with PBS, counterstained in hematoxylin, blued in running water, dehydrated with gradient alcohol, cleared with xylene, and mounted with neutral gum. Areas positive for SP or CGRP in randomly selected fields of vision (original magnification ×200) were counted using Image-Pro Plus 6.0 (Media Cybernetics, Rockville, MD, USA). The resulting data were collected as immunohistochemical staining-positive signal cumulative optical density (IOD) values, which represent the average positive area. These IOD values were used as quantitative measures of tissue expression levels.

### 2.6. Statistical Analysis

**Figure 1:**
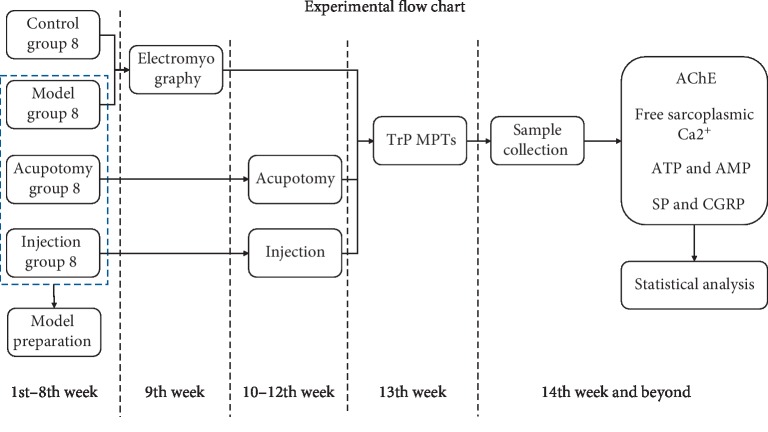
The flow chart shows procedures applied to the animals in timeline. Model preparation was in the 1st–8th week. Electromyography detection was in the 9th week. Acupotomy and injection were conducted in the 10–12th week. MPT was detected in the 13th week. Sample collection and other tests were in the 14th week and beyond.

Data were analyzed using the SPSS 22.0 statistical software (v22.0; IBM Corp., Armonk, NY, USA). For normally distributed data with homogeneous variance, analysis of variance (ANOVA) was used to compare four groups. For non-normally distributed data, the Kruskal–Wallis test was used. All data are expressed as mean ± standard deviation. *P* < 0.05 and *P* < 0.01 were considered to indicate significant and very significant differences, respectively (Figure 1).

## 3. Results

### 3.1. Electromyography of Model Group

**Figure 2:**
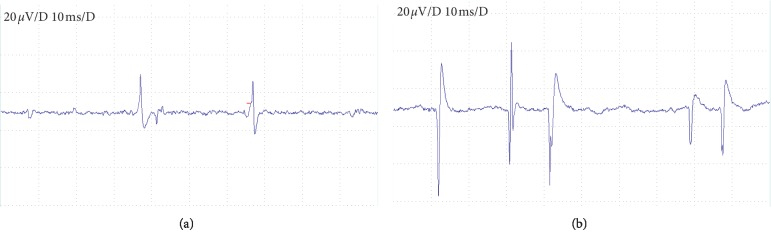
(a) The typical endplate potential, which turns negative from baseline. (b) The fibrillation potential, which is positive from baseline and then turns negative. The scanning speed and sensitivity are 10 ms/D and 20 *μ*V/D, respectively.

Electromyography was recorded at 10 ms/D and 20 *μ*V/D.  Figure 2(a) shows a representative typical endplate potential, which turns negative from baseline.  Figure 2(b) shows the fibrillation potential, which turns positive from baseline and then turns negative. This suggests the presence of TrPs in the vastus medialis of the model group rats [6].

### 3.2. Acupotomy Effect on TrP MPTs

**Figure 3:**
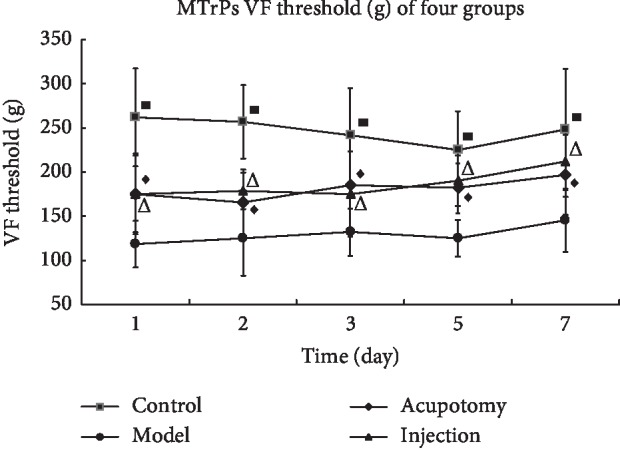
MPT changes (g) in each group (control, model, acupotomy, and injection; *n* = 8 per group) determined by the Von Frey mechanical pain stimulator test. ^■^*P* < 0.05 versus the model group. ^Δ^*P* < 0.05 versus the model group. ^♦^*P* < 0.05 versus the model group. The error bars represent standard deviations.

Compared with the control group, the MPT of the model group decreased on the 1st, 2nd, 3rd, 5th, and 7th days after intervention (*P* < 0.05, Figure 3). Moreover, compared with the model group, the MPT increased in the acupotomy group on the 1st, 2nd, 3rd, 5th, and 7th days after intervention (*P* < 0.05, Figure 3). Further, relative to the model group, the MPT was higher in the lidocaine injection group on the 1st, 2nd, 3rd, 5th, and 7th days after intervention (*P* < 0.05, Figure 3). Comparisons between the acupotomy and injection groups showed no significant difference in MPTs at any time point tested (*P* > 0.05, Figure 3).

### 3.3. Acupotomy Effect on AChE

**Figure 4:**
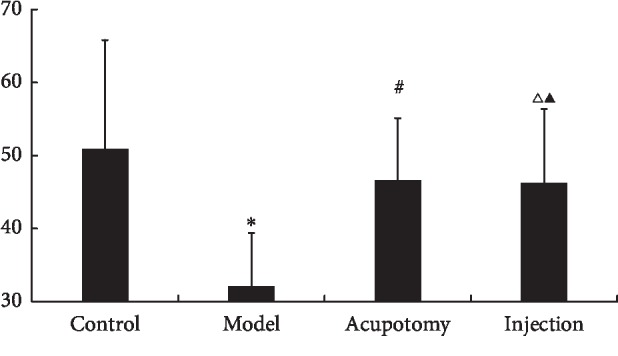
AChE concentrations (pg/mg) in each group (control, model, acupotomy, and injection; *n* = 8 per group). ^*∗*^*P* < 0.05 compared with the control group, ^#^*P* < 0.05 compared with the model group, ^Δ^*P* < 0.05 compared with the model group, and ^▲^*P* > 0.05 compared with the acupotomy group. The error bars represent the standard deviations.

AChE levels were significantly lower (*P* < 0.05) in the model group than the control group (*P* < 0.05). However, AChE levels in the acupotomy (*P* < 0.05) and injection (*P* < 0.05) groups were significantly increased compared with the model group (Figure 4, Table 1). There was no significant difference between the acupotomy and injection groups (*P* > 0.05, Figure 4, Table 1). These data show that acupotomy can increase AChE levels in TrPs.

### 3.4. Acupotomy Effect on Free Sarcoplasmic Ca^2+^

**Figure 5:**
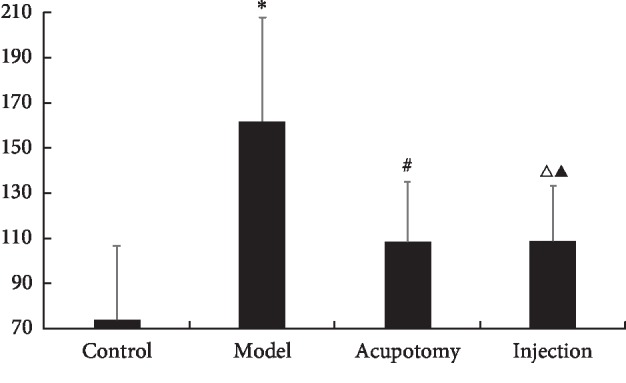
Free sarcoplasmic Ca^2+^ concentrations (nmol/L) in each group (control, model, acupotomy, and injection; *n* = 8 per group). ^*∗*^*P* < 0.01 compared with the control group, ^#^*P* < 0.05 compared with the model group, ^Δ^*P* < 0.05 compared with the model group, and ^▲^*P* > 0.05 compared with the acupotomy group. The error bars represent the standard deviations.

Free sarcoplasmic Ca^2+^ in the model group was significantly higher than in the control group (*P* < 0.05). Relative to the model group, free sarcoplasmic Ca^2+^ in the acupotomy and injection groups was decreased significantly (both *P* < 0.05, Figure 5, Table 2). There was no significant difference between the acupotomy and injection groups (*P* > 0.05, Figure 5, Table 2). These findings demonstrate that acupotomy can reduce free sarcoplasmic Ca^2+^ levels in TrPs.

### 3.5. Acupotomy Effect on ATP and AMP

**Figure 6:**
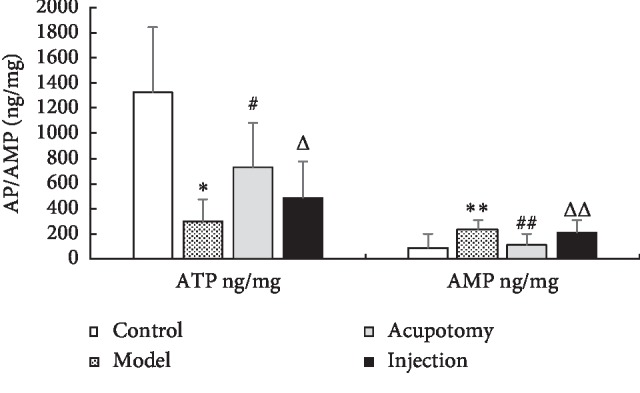
ATP and AMP levels (ng/mg) in each group (control, model, acupotomy, and injection; *n* = 8 per group). ^*∗*^*P* < 0.05 for ATP levels compared with the control group, ^#^*P* < 0.05 for ATP levels compared with the model group, ^Δ^*P* > 0.05 for ATP levels compared with the model group, ^*∗∗*^*P* < 0.05 for AMP levels compared with the control group, ^##^*P* < 0.05 for AMP levels compared with the model group, and ^ΔΔ^*P* > 0.05 for AMP levels compared with the model group. The error bars represent the standard deviations.

ATP levels in the model group were significantly lower than those in the control group (*P* < 0.05). Relative to the model group, ATP increased significantly in the acupotomy group (*P* < 0.05, Figure 6, Table 3); however, there was no significant difference between the model and injection groups (*P* > 0.05, Figure 6, Table 3). These findings indicate that acupotomy can enhance ATP levels in TrPs.

AMP levels in the model group were significantly higher than those in the control group (*P* < 0.05), but those in the acupotomy group were significantly lower than those in the model group (*P* < 0.05, Figure 6, Table 3). Again, there was no significant difference between the model and injection groups (*P* > 0.05, Figure 6, Table 3). These results show that acupotomy can decrease AMP levels in TrPs.

### 3.6. Acupotomy Effect on SP and CGRP

**Figure 7:**
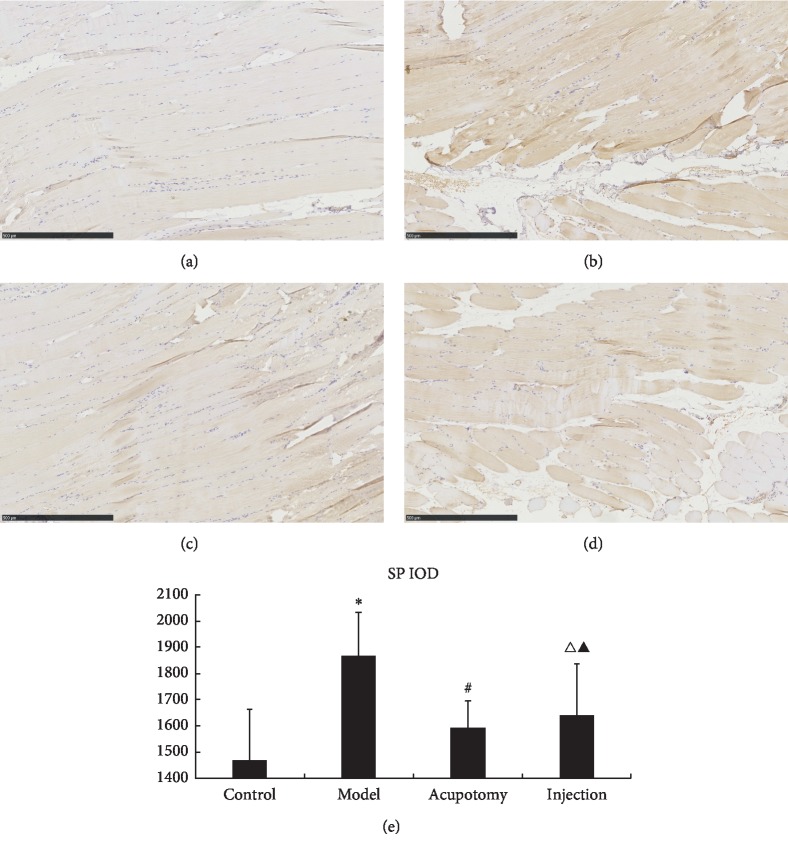
Immunohistochemistry for SP (light microscopy, ×200). SP expression was yellowish brown in muscle fibers. SP and CGRP levels by group (control, model, acupotomy, and injection; *n* = 8 per group). ^*∗*^*P* < 0.05 for SP compared with the control group; ^#^*P* < 0.05 for SP compared with the model group; ^Δ^*P* < 0.05 for SP compared with the model group; ^▲^*P* > 0.05 for SP compared with the acupotomy group. The error bars represent the standard deviations. (a) Control group. (b) Model group. (c) Acupotomy group. (d) Injection group. (e) SP IOD.

In Figure 7, SP expression was yellowish brown in muscle fibers. SP levels were significantly higher in the model group than the control group (*P* < 0.05), but they were significantly lower in the acupotomy group than the model group (*P* < 0.05, Figure 6, Table 4). Furthermore, compared with the model group, SP levels were significantly lower in the injection group (*P* < 0.05, Table 4). There was no significant difference between the acupotomy and injection groups (*P* > 0.05, Figure 7, Table 4). Hence, our findings indicate that acupotomy can reduce SP levels in TrP muscle tissue.

**Figure 8:**
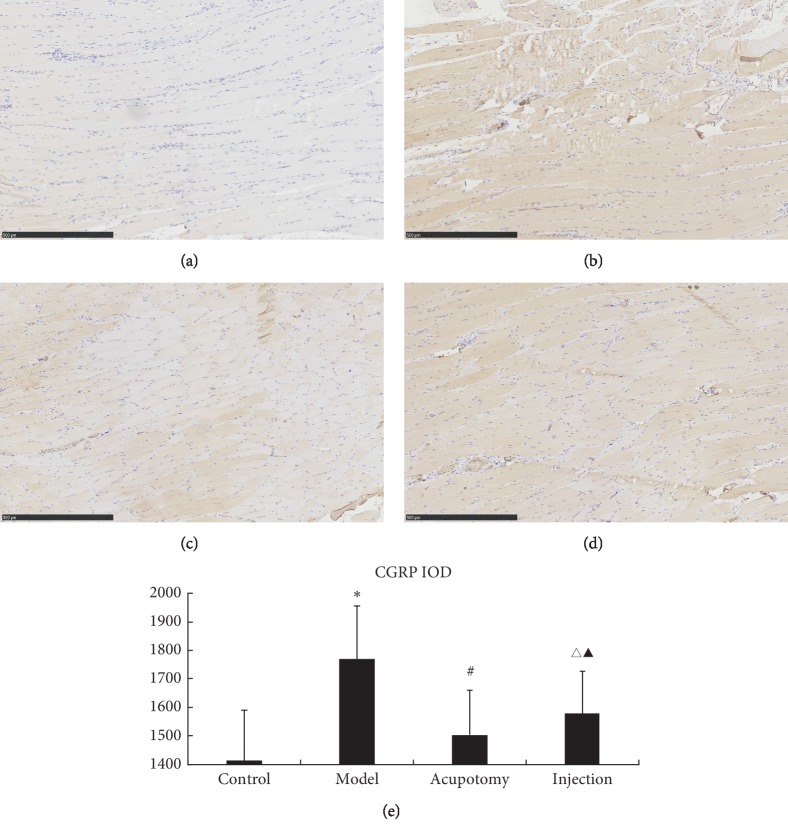
Immunohistochemistry for CGRP (light microscopy, ×200). CGRP expression was yellowish brown in muscle fibers. CGRP levels were significantly higher in the model group than the control (*P* < 0.05), acupotomy (*P* < 0.05), and injection (*P* < 0.05) groups. There was no significant difference in CGRP content between the acupotomy and injection groups (*P* > 0.05). SP and CGRP levels by group (control, model, acupotomy, and injection; *n* = 8 per group). ^*∗*^*P* < 0.05 for CGRP compared with the control group; ^#^*P* < 0.05 for CGRP compared with the model group; ^▲^*P* > 0.05 for CGRP compared with the acupotomy group; ^Δ^*P* < 0.05 for CGRP compared with the model group. The error bars represent the standard deviations. (a) Control group. (b) Model group. (c) Acupotomy group. (d) Injection group. (e) CGRP IOD.

In Figure 8, CGRP expression was yellowish brown in muscle fibers. CGRP levels were significantly higher in the model group than the control group (*P* < 0.05), but those in the acupotomy (*P* < 0.05) and injection (*P* < 0.05) groups were significantly lower than those in the model group. There was no significant difference in CGRP content between the acupotomy and injection groups (*P* > 0.05, Figure 8, Table 4). These results suggest that acupotomy can reduce CGRP content in TrP muscle tissue.

## 4. Discussion

The results of this study demonstrate that the MPT, AChE, free sarcoplasmic Ca^2+^, ATP, AMP, SP, and CGRP levels were altered in rats after TrP model generation, and acupotomy improved these effects. Other than the changes in ATP and AMP levels, which were improved by acupotomy but not lidocaine injection, there were no significant differences between the effects of acupotomy and those of lidocaine injection.

### 4.1. Energy Crisis at Trigger Points

Despite several theories, the exact nature of TrPs remains unknown [11, 12]; however, electrophysiological and histological evidence support the integrated hypothesis of the cause of TrPs [13], which contends that an abnormal increase in ACh causes increased energy consumption in TrPs. Specifically, motor endplates in active TrPs release excessive ACh in the resting state, resulting in continuous depolarization of muscle fibers. This causes the sarcoplasmic reticulum to release excessive Ca^2+^ into myoplasm, followed by continuous muscle fiber contraction that consumes excessive energy. Inadequate blood flow due to continuous contraction of muscle fibers cannot effectively replenish energy and oxygen. In energy crises, Ca^2+^ pumps in the sarcoplasmic reticulum fail to reduce the free Ca^2+^ in myoplasm, aggravating muscle fiber contraction. Energy crises increase algogenic substances at local levels, which further perpetuates Ach release at motor endplates, creating a vicious circle [14].

### 4.2. AChE Indicates Endplate Function

This neuromuscular junction is responsible for passing nerve impulses to the skeletal muscles, causing muscle contractions. When an action potential reaches the presynaptic terminal of a motor neuron, ACh is released into the synaptic space where it binds to nicotinic ACh receptors (AChRs) on the muscle membrane. Binding of ACh to an AChR depolarizes the muscle fibers. ACh can be hydrolyzed by AChE, which is the main factor controlling the duration of ACh effects. Therefore, AChE measurement is often used to indirectly assess ACh levels.

Excessive ACh at the endplate, induced by diisopropylfluorophosphate, leads to the formation of the contracture TrP nodules [15], while injection of botulinum toxin into TrPs in New Zealand rabbits can prevent ACh release into the synaptic space, thus reducing endplate noise levels in myofascial trigger spot regions [16]. In addition, TrP pathogenesis is related to the sympathetic nervous system [17] through regulation of motor nerve synaptic vesicle release and levels of postsynaptic membrane AChR via its effects on the Gαi_2_-Hdac_4_-Myogenin-MuRF_1_ pathway [18].

In this study, AChE levels were decreased in the TrP model compared with control and acupotomy or lidocaine increased AChE levels. Similarly, a previous investigation reported that ACh and AChR levels were decreased after dry needling at precise TrPs, but AChE was increased [19]. Overall, the evidence suggests that acupotomy or dry needle treatment may affect TrPs through regulation of AChE levels in neuromuscular junctions.

In addition to inactivating ACh, AChE also promotes neuromuscular junction survival [20]. In addition to reflecting the functional state of motor neurons [21], AChE activity can also indicate endplate degeneration and regeneration [22, 23]. AChE activity is markedly decreased in denervated skeletal muscle, and the presence of AChE can be considered a marker of nerve fiber regeneration. Hence, the decrease in AChE in TrPs may indicate that TrPs are associated with changes in nerve or endplate function. Indeed, neuroaxonal degeneration and neuromuscular transmission are disordered in TrP-containing muscles [24]. In addition, the type of muscle excitation can also regulate AChE activity in the motor endplate, suggesting that the type of synaptic transmission can also regulate AChE [25]. How continuous contraction of muscle fibers at TrPs is related to decreased AChE levels warrants further study.

### 4.3. Two Possible Reasons for Regulating Free Sarcoplasmic Ca^2+^

Ca^2+^ is a chemical signal that can communicate messages within cells [26]. Originating from motor endplates at myofascial TrPs, spontaneous electrical activity (SEA) can reflect muscle fiber excitability, depending on TrP sensitivity [27]. Decreased AChE activity increases the amplitude of miniature excitatory postsynaptic currents, prolonging their attenuation time and causing sustained AChR activation that induces a Ca^2+^ influx [28]. Increases and decreases in free sarcoplasmic Ca^2+^ directly influence skeletal muscle fiber contraction and relaxation, and one important characteristic of TrPs is their continuous contraction that causes taut band formation. The Ca^2+^ channel blocker verapamil can effectively inhibit SEA in myofascial trigger spots in rabbit biceps femoris muscles [29]. Verapamil prevents Ca^2+^ influx through voltage-dependent Ca^2+^ channels on the plasma membrane, and it removes the inhibition of sodium (Na^+^) pumps caused by increased Ca^2+^ loads, thereby reducing intracellular Ca^2+^ and ensuring normal physiological cell function. This fully shows that intracellular Ca^2+^ content can have a significant impact on the trigger point.

This investigation provides direct proof that free sarcoplasmic Ca^2+^ concentrations in the model group were significantly higher than those in the control group. Furthermore, acupotomy effectively reduced free sarcoplasmic Ca^2+^ in TrP muscle cells. The reason why acupotomy reduces free sarcoplasmic Ca^2+^ requires further study, but it may be associated with changes in AChE and ACh and/or it may be related to transient muscle cell Ca^2+^ levels.

Firstly, ACh release is related with muscle spindle. Stecco et al. [30] believe that if the fascia outside the muscle fiber is denser, it will hinder the shortening of the muscle spindle and even produce a chronic stretch on the muscle spindle, thus causing the continuous excitement of the muscle spindle. This explains the increased ACh from endplates at the trigger point and the continued muscle fibers contraction. Acupotomy or dry needling may reduce the excitability of muscle spindles by reducing fascia tension and ultimately reducing the ACh release and intracellular Ca^2+^.

Secondly, Na^+^-Ca^2+^ exchanger deserves attention. The Na^+^-Ca^2+^ exchanger NCX is an ATP-independent bidirectional transporter with two working modes. Hypoxia leads to decreased Na^+^/potassium (K^+^)-ATPase activity and increased intracellular Na^+^ concentrations, causing NCX-mediated exchange of Ca^2+^ into cells and consequent Ca^2+^ overload and damage. In a study of the effects of acupuncture on Ca^2+^ in skeletal muscle cells, Zhuqing et al. inferred that local acupuncture for delayed onset muscle soreness may affect NCX function, thereby inhibiting cytoplasmic Ca^2+^ overload [31].

### 4.4. ATP and ADP Indicate Energy Supply

The persistent contraction of muscle fiber TrPs increases local energy consumption and restricts blood circulation. ATP produced by oxidative phosphorylation is the direct source of energy for muscle contraction and sarcoplasmic reticulum Ca^2+^ reabsorption. A lack of ATP in the muscle reduces its ability to function, including both contraction and relaxation. Adenosine 5′-diphosphate (ADP) and AMP are degradation products of ATP and have important functions in fatigue [32, 33]. In this study, ATP levels were lower in the model group than control group, which is consistent with the integrated hypothesis. Furthermore, this decrease was attenuated following acupotomy. This effect may be caused by improved blood circulation in TrPs in response to decreased free sarcoplasmic Ca^2+^.

### 4.5. CGRP Enhances ACh Effect at Motor Endplate

Continuous contraction leads to local ischemia and hypoxia in TrPs, which can cause the tissue to release vasoactive substances that act on nociceptors, leading to nerve sensitization, pain, and Ach release from nerve endings [34]. Using microanalytical techniques, Shah et al. found that concentrations of protons, bradykinin, CGRP, SP, tumor necrosis factor-alpha, interleukin-1 beta, serotonin, and norepinephrine were significantly higher in active TrPs [6, 35, 36]. Muscle pain is associated with muscle nociceptor activation by a variety of endogenous substances including neuropeptides and inflammatory mediators. SP and CGRP can cause plasma extravasation, inflammation, and nociceptive effects [6]. CGRP exists in the ends of ɑ motor neurons and promotes ACh release into the synaptic cleft [15]. Importantly, in relation to TrPs, CGRP increases the number of AChRs and inhibits AChE activity at motor endplate [37, 38], thereby enhancing the effect of ACh at the motor endplate. Gerwin and Shah hypothesized that CGRP enhances the motor endplate response to ACh by increasing the activity and synthesis of AChRs [39]. In this investigation, we found significantly elevated levels of SP and CGRP in tissue from TrPs. Furthermore, both SP and CGRP were significantly lower in the acupotomy group than the model group, which is consistent with the common observation of decreased pain after TrP treatment. Acupuncture increases local nitric oxide and microcirculation [40, 41], so local microcirculation in TrPs should be evaluated before and after needling to determine whether it dilutes algogenic substances.

In Figures 7 and 8, images of acupotomy and injection groups show lymphocyte infiltration and destruction of the fiber order, which may indicate the healing process of injuries. Because both acupotomy and injection are invasive, is the healing process of injuries related to treatment effect? Stecco et al. [30] believe that the increased fascia density outside the muscle fiber may excite muscle spindle, causing increased ACh from endplates and continued contraction. Acupotomy or dry/wet needling may reduce the excitability of muscle spindles by changing the inner environment around the fibers.

### 4.6. Relationship between TrP and Ashi Points

Herman et al. proposed the concept of TrPs in 1942 [42]. Since accepting the concept of TrPs, some Chinese scholars believe that TrPs and TCM ashi points exhibit considerable overlap in their positions, needling sensation, and indications [43]. When investigations into TrPs began, TCM practitioners believed that TrPs can be considered typical representative ashi points. But a contrary opinion is that the overlap of a number of pain points does not change that basic difference between acupoints and TrPs. Acupoints and TrPs are derived from vastly different concepts [44]. No matter the two concepts have similarities or differences in essence, it will be an hint to promote the reseach of ashi points in TCM. Although ashi points have been understood by practitioners of TCM for several centuries and various acupuncture methods have been applied in TCM to treat ashi points, deep research into ashi points has been lacking.

## 5. Conclusion

Developed from ancient acupuncture techniques, acupotomy is similar to dry needling; both require accurate insertion of needles into local TrPs, and they may also work via similar mechanisms. The observations from our study support the hypothesis that energy crises affect TrP muscle fibers. Furthermore, our data demonstrate that acupotomy can influence TrP MPTs and levels of AChE, free sarcoplasmic Ca^2+^, ATP, AMP, SP, and CGRP. Its effects were similar to local lidocaine injection, except only acupotomy affected ATP and AMP levels. It is indicated that acupotomy has effect on trigger point pathophysiology, which is different from acupuncture analgesia. Future research will focus on the mechanism by which acupotomy resolves energy crises in TrPs, peripheric or central?

The general understanding of acupuncture mechanism is that the mechanical signals generated by needle insertion are converted into nerve signals through the acupoint and transmitted to the central nervous system and on to the efferent system and effector organs via the neuro-endocrine-immune system [45]. Further, acupuncture may have peripheral effects, independent of overall neurohumoral regulation [46, 47]. Zhuqing et al. [48] found that local acupuncture is effective in delaying muscle soreness onset because it adjusts the catabolism and anabolism of muscle contractile proteins. This differs from the mechanism involved in acupuncture analgesia. The same effect was observed in isolated semitendinosus muscle experiments, suggesting that acupuncture may have a peripheral mechanism independent of overall neurohumoral regulation. For visceral disorders, it is necessary for acupuncture to work via the neuro-endocrine-immune system because the viscera cannot be needled directly. But for myofascial lesions, it is different because muscular fasciae can be needled directly. For myofascial disorder, what will happen when it is needled directly? Acupuncture was shown to trigger a local increase in adenosine in human subjects [49, 50] and to increase local microcirculation [40, 41]. It is still controversial whether a trigger point is a central or peripheral phenomenon [11]. Accurate positioning at TrPs is an important requirement for both acupotomy and dry needling, suggesting that the effects of these procedures may have similar underlying peripheral mechanisms; however, a review reported that due to the scarcity of reliable studies, the current evidence on the local effects of acupuncture is insufficient to draw reliable conclusions [51]. Additional investigations are needed to verify the local effects of acupuncture.

The limitation of this study is that only one method was used to test each variable; therefore, our data lack verification with multiple experimental methods. And several anesthesia procedures during the experiment may influence the results.

## Figures and Tables

**Table 1:** Changes in AChE levels after acupotomy ($\bar{x}\pm s$).

Groups	*N*	AChE (pg/mg)
Control	8	50.91 ± 14.88
Model	8	32.18 ± 7.27^*∗*^
Acupotomy	8	46.70 ± 7.88^#^
Injection	8	46.35 ± 9.99^▲Δ^

Versus control group: *P*^*∗*^ < 0.05; versus model group: *P*^#^ < 0.05; versus model group: *P*^Δ^ < 0.05; versus acupotomy group: *P*^▲^ > 0.05.

**Table 2:** Changes in free sarcoplasmic Ca^2+^ levels after acupotomy ($\bar{x}\pm s$).

Groups	*N*	Free sarcoplasmic Ca^2+^ (nmol/L)
Control	8	74.02 ± 32.71
Model	8	161.81 ± 46.14^*∗*^
Acupotomy	8	105.52 ± 26.52^#^
Injection	8	109.1 ± 24.41^▲Δ^

Versus control group: *P*^*∗*^ < 0.05; versus model group: *P*^#^ < 0.05; versus model group: *P*^Δ^ < 0.05; versus acupotomy group: *P*^▲^ > 0.05.

**Table 3:** Changes in ATP/AMP levels after acupotomy ($\bar{x}\pm s$).

Groups	*N*	ATP (ng/mg)	AMP (ng/mg)
Control	8	1322.15 ± 516.51	85.60 ± 114.29
Model	8	298.34 ± 181.82^*∗*^	239.18 ± 71.05^*∗∗*^
Acupotomy	8	734.62 ± 344.53^#^	108.76 ± 89.89^##^
Injection	8	490.73 ± 280.36^Δ^	208.49 ± 101.65^ΔΔ^

Versus control group: *P*^*∗*^ < 0.05; versus model group: *P*^#^ < 0.05; versus model group: *P*^Δ^ > 0.05; versus control group: *P*^*∗∗*^ < 0.05; versus model group: *P*^##^ < 0.05; versus model group: *P*^ΔΔ^ > 0.05.

**Table 4:** Changes in SP/CGRP IOD after acupotomy ($\bar{x}\pm s$).

Groups	*N*	SP	CGRP
Control	8	1468 ± 195.24	1412.63 ± 178.38
Model	8	1866.38 ± 165.38^*∗*^	1770.3 ± 186.90^*∗∗*^
Acupotomy	8	1591.25 ± 102.97^#^	1502.38 ± 156.35^##^
Injection	8	1640.75 ± 197.48^Δ^	1578.13 ± 148.58^ΔΔ^

Versus control group: *P*^*∗*^ < 0.05; versus model group: *P*^#^ < 0.05; versus model group: *P*^Δ^ < 0.05; versus acupotomy group: *P*^▲^ > 0.05; versus control group: *P*^*∗∗*^ < 0.05; versus model group: *P*^##^ < 0.05; versus model group: *P*^ΔΔ^ < 0.05; versus acupotomy group: *P*^▲▲^ > 0.05.

## Data Availability

The data used to support the findings of this study are available from the corresponding author upon request.
